# Understanding Etiopathogenesis and Clinical Outcomes in Acute Pancreatitis: An Experience From a Tertiary Care Teaching Hospital in Andhra Pradesh

**DOI:** 10.7759/cureus.87913

**Published:** 2025-07-14

**Authors:** Chandra Shekar Kali, Vinoda Kadali, Sandeep Chakra G, Tarun Kumar Suvvari, Sindhuja Karangula, Srinivasa Rajasekhar Kata, Nagateja Yedida, Ramya Sree Muppavarapu, Sumanth Gundraju

**Affiliations:** 1 General Medicine, Government Medical College, Rajamahendravaram, IND; 2 General Medicine, Rangaraya Medical College, Kakinada, IND; 3 Internal Medicine, Government Medical College, Paderu, IND; 4 Internal Medicine, Rangaraya Hospital, Kakinada, IND; 5 Research, Squad Medicine and Research (SMR), Visakhapatnam, IND; 6 Internal Medicine, Malla Reddy Institute of Medical Sciences, Hyderabad, IND; 7 Medicine, Katuri Medical College and Hospital, Guntur, IND; 8 Medicine, Rangaraya Medical College, Kakinada, IND; 9 Internal Medicine, Konaseema Institute of Medical Sciences, Vijayawada, IND

**Keywords:** acute pancreatitis, bisap score, clinical outcome, clinical study, ct severity index, prognostic scoring

## Abstract

Introduction

Acute pancreatitis (AP) is a potentially life-threatening inflammatory disorder of the pancreas with a wide spectrum of clinical manifestations. Early identification of disease severity is critical for guiding management and improving outcomes. Prognostic scoring systems such as the Bedside Index for Severity in Acute Pancreatitis (BISAP) and the CT severity index (CTSI) are commonly used to predict disease progression and complications. This study aimed to evaluate the clinical profile of patients with AP and assess the utility of BISAP and CTSI scores in predicting clinical outcomes.

Methods

This cross-sectional study was conducted in the Department of General Medicine from April 2025 to May 2025 (two months). Patients diagnosed with AP were enrolled based on clinical, biochemical, and radiological criteria. Detailed clinical histories, physical examinations, and laboratory investigations were recorded. BISAP scores were calculated at admission, and contrast-enhanced CT scans were performed on day four or five of illness to determine CTSI scores. Patients were monitored throughout their hospital stay, and outcomes were assessed at discharge.

Results

Fifty patients with AP were enrolled. The most common etiology was alcohol consumption (74%). A BISAP score ≥3 was observed in 6% of patients and was significantly associated with mortality (p=0.039). The CTSI indicated severe AP in 12% of patients and was significantly associated with both mortality (p=0.002) and complications (p=0.002). The BISAP score demonstrated excellent predictive ability for mortality [area under the curve (AUC)=0.934], while CTSI provided superior sensitivity and predictive accuracy for complications (AUC=0.658). A moderate positive correlation was observed between BISAP and CTSI (r=0.45, p=0.019).

Conclusions

Both BISAP and CTSI are effective predictors of outcomes in AP. BISAP is a valuable bedside tool for early mortality risk stratification, particularly in resource-limited settings, while CTSI remains indispensable for assessing complications and guiding interventional strategies. The combined use of both scoring systems can enhance clinical decision-making and optimize patient management.

## Introduction

Acute pancreatitis (AP) is a sudden inflammatory condition of the pancreas with a wide spectrum of clinical presentations, ranging from mild, self-limiting episodes to severe forms with life-threatening complications [1]. While mild cases can often be managed conservatively with intravenous fluids and bowel rest, severe cases may necessitate admission to the ICU and even surgical intervention. AP is diagnosed when at least two of the following three criteria are present: acute onset of characteristic abdominal pain, elevated serum amylase or lipase levels (lipase > amylase), and imaging findings consistent with pancreatitis [2,3]. Clinically, it can manifest with hypotension, systemic inflammatory response, metabolic disturbances, sepsis, multiorgan failure, and in some cases, even death. Although the overall mortality from AP has declined, from approximately 12% to 2%, the rates remain significantly high among patients with severe disease [4-6].

The global incidence of AP ranges from 13 to 45 per 100,000 population and has been increasing steadily over the past two decades [6]. Understanding the etiology of AP is crucial, not only for guiding immediate clinical management but also for preventing recurrence. Recurrence rates can be as high as 50% in alcohol-induced AP and 32-61% in untreated gallstone-related cases [7]. Despite advancements in intensive care and management protocols, morbidity and mortality associated with severe AP remain high. Therefore, early etiological identification and severity stratification are essential. Various prognostic scoring systems have been developed since the 1970s. Among them, the Balthazar CT severity index (CTSI) and the Bedside Index for Severity in Acute Pancreatitis (BISAP) are widely used. While CTSI offers radiological grading, BISAP is a simpler, clinically applicable tool with good predictive value for in-hospital mortality [8,9].

This study was undertaken to evaluate the clinical spectrum and outcomes of patients diagnosed with AP, with a specific focus on identifying current patterns in clinical presentation and disease severity. It also aims to assess the prognostic performance of two widely used scoring systems - BISAP and CTSI - in predicting patient outcomes. By correlating these scoring tools with clinical progression and severity, the study seeks to enhance early risk stratification and aid in more informed management decisions in AP.

## Materials and methods

This cross-sectional study was conducted in the Department of General Medicine at a tertiary care teaching hospital from April 2025 to May 2025 (two months). A total of 50 patients aged 18 years and above, presenting with acute abdominal pain and diagnosed with AP based on clinical presentation and biochemical evidence (serum amylase and/or lipase levels elevated to more than three times the normal range), were included. Patients with other causes of acute abdomen, such as appendicitis, gastritis, cholecystitis, bowel perforation, myocardial infarction, and renal calculi, as well as those with CT evidence of chronic pancreatitis (e.g., pancreatic calcifications), were excluded. Ethical approval was obtained from the Institutional Ethics Committee, and informed written consent was collected from all participants.

Upon enrollment, patients underwent a detailed clinical assessment using a semi-structured questionnaire that included demographic details, medical history, substance use, and medication history. Vital signs were recorded, and a physical examination was performed. BMI was calculated, and neurological status was evaluated using the Glasgow Coma Scale (GCS) [10]. All patients underwent routine investigations including complete blood count, liver and renal function tests, serum electrolytes, ECG, chest X-ray, and abdominal ultrasonography. Hematological parameters were assessed using the ABX Micros 60 analyzer (HORIBA Advanced Techno, Co., Ltd, Kyoto, Japan). Contrast-enhanced CT (CECT) of the abdomen was performed between the fourth and fifth day of illness to determine the CTSI [11,12]. The BISAP score, ranging from 0 to 5, was calculated for each patient based on clinical, biochemical, and radiological parameters at admission [13]. Patients were managed as per hospital protocols and were observed throughout their hospital stay to assess outcomes, including recovery or mortality. Cases with suspected obstructive etiology were referred to the surgical department for further management.

Data were analyzed using SPSS Statistics version 20 software (IBM Corp., Armonk, NY). Categorical variables were evaluated using the Chi-square test. The predictive abilities of the BISAP and CTSI scores were assessed using positive and negative predictive values. Receiver operating characteristic (ROC) curves were generated to compare the diagnostic accuracy of the two scoring systems. All statistical analyses were performed with a significance level set at p<0.05. All original scoring systems and measurement instruments used have been cited above. The semi-structured questionnaire was approved by the Institutional Ethics Committee; no external permissions for proprietary instruments were required as no such instruments were used.

## Results

A total of 50 patients were enrolled in the study. The mean age was 38 ± 11 years (range: 18-65 years). Age-stratified analysis revealed that only 6% (n=3) of patients were aged 60 years or older, indicating that the majority (94%) were under 60 years of age. The most commonly affected age group was 20-39 years, comprising 56% (n=28) of the study population. In terms of gender distribution, males were overwhelmingly predominant, accounting for 90% (n=45) of cases, while females represented only 10% (n=5), yielding a male-to-female ratio of 9:1. Alcohol emerged as the predominant etiological factor for AP, accounting for 74% (n=37) of the cases. A history of alcohol consumption was documented in 84% (n=42) of patients, among whom five had additional risk factors, including gallstones (n=1), drug use (n=3), and prior abdominal surgery for gastric malignancy (n=1). The association between alcohol and AP was significantly higher among male patients, whereas gallstone-induced pancreatitis was more frequently observed in females. Biliary pancreatitis was identified in 6% (n=3) of cases. Idiopathic pancreatitis accounted for 10% (n=5), and 2% (n=1) were solely drug-induced causes. One patient had a history of abdominal surgery for gastric cancer. In 10% (n=5) of patients, multiple contributory factors were identified, indicating a multifactorial etiology.

Regarding clinical presentation, abdominal pain was the most common presenting symptom, reported in 98% (n=49) of patients. Nausea and vomiting were observed in 68% (n=34) and 62% (n=31) of cases, respectively, with one patient (2%) experiencing hematemesis. Abdominal distension was noted in 28% (n=14) of patients. Fever and jaundice were present in 10% (n=5) and 12% (n=6) of cases, respectively. Altered bowel habits were reported by 20% (n=10) of patients; of these, eight had constipation, two had increased stool frequency, and one presented with melena. Breathlessness and reduced urine output, indicative of potential complications such as acute respiratory distress syndrome (ARDS) and acute kidney injury (AKI), were observed in 12% (n=6) and 8% (n=4) of cases, respectively. Among the 50 patients studied, 25 (50%) presented with acute abdominal pain for the first time, while the remaining 25 (50%) had reported a similar episode in the past. Of those with prior episodes, 10 had been previously diagnosed with AP, whereas in 15 patients, the underlying cause remained undetermined. This indicates a recurrence rate of 20% for AP among the study participants. Additionally, two patients (4%) had a known history of gallstones and had received medical but not surgical management. Comorbid conditions included diabetes mellitus in three patients (6%) and hypertension in seven patients (14%), all of whom were undergoing regular treatment.

Among the 50 patients, 84% (n=42) reported regular alcohol consumption and 74% (n=37) had a history of smoking. Most patients (88%) had a normal BMI, while 10% were overweight and 2% were underweight. Notably, three of the five overweight patients succumbed to the illness. The GCS score was 15/15 in 48 patients; the remaining two had lower scores (13 and 14), and both died. The mean systolic and diastolic blood pressures were 120 ± 20 mmHg and 78 ± 13 mmHg, respectively. Tachycardia (pulse >90 bpm) was present in 32% of cases, tachypnea (RR >20) in 18%, and hyperthermia (≥38°C) in 10%. These findings reflect the systemic inflammatory response often seen in moderate to severe AP. On abdominal examination, tenderness was observed in all 50 patients (100%). Guarding was present in nine patients (18%), abdominal distension in 15 (30%), and ascites in 11 (22%). No cases showed evidence of ecchymosis or palpable abdominal mass. Auscultation revealed normal bowel sounds in 29 patients (58%), sluggish sounds in 18 (36%), and absent bowel sounds in three patients (6%), indicating varying degrees of gastrointestinal motility impairment.

Investigation workup

All 50 patients underwent comprehensive laboratory and radiological investigations. The mean (±SD) serum amylase level was 606 ± 429 IU/L (range: 200 to 2173 IU/L), while the mean serum lipase level was 617 ± 366 IU/L (range: 204 and 1628 IU/L - both exceeding the diagnostic threshold of three times the normal value in all patients). The mean random blood sugar (RBS) level was 113 ± 49.9 mg/dL (range: 59-284 mg/dL). Blood urea nitrogen (BUN) levels varied widely, with a mean of 18.25 ± 20.16 mg/dL (range: 9.3-149.8 mg/dL), and serum creatinine values ranged from 0.4 to 14 mg/dL, with a mean of 1.3 ± 2.1 mg/dL. Total leukocyte count (TLC) had a mean of 8471 cells/cumm, reflecting variable systemic responses to inflammation. Mean serum bilirubin was 1.4 ± 1.1 mg/dL, and mean serum calcium was 8.7 ± 0.9 mg/dL. Serum triglycerides were also evaluated, with a mean value of 140 ± 46.2 mg/dL, ranging from 62 to 266 mg/dL. Biochemical parameters of patients with AP are summarized in Table 1.

**Table 1:** Biochemical Parameters of Patients With Acute Pancreatitis

Parameter	Mean	Standard Deviation	Minimum	Maximum
Serum Amylase (IU/L)	606.06	429.09	200	2173
Serum Lipase (IU/L)	616.86	365.68	204	1628
Random Blood Sugar (mg/dL)	113.00	49.90	59	284
Blood Urea Nitrogen (mg/dL)	18.25	20.16	9.33	149.8
Serum Creatinine (mg/dL)	1.30	2.10	0.4	14.0
Total Leukocyte Count (cells/cumm)	8471	2036	4900	15200
Total Serum Bilirubin (mg/dL)	1.40	1.10	0.6	6.5
Serum Calcium (mg/dL)	8.72	0.86	6.4	11.7
Serum Triglycerides (mg/dL)	140.00	46.20	62	266

In addition to biochemical evaluation, ultrasonography of the abdomen was performed in all patients. A bulky pancreas was noted in 72% (n=38) of cases, consistent with acute inflammation. Altered echotexture of the pancreas was observed in 38% (n=19), while peripancreatic inflammation was evident in 4% (n=2). In 18% (n=9) of patients, the pancreas could not be visualized adequately due to a poor acoustic window. Ascites was present in 30% (n=15), pleural effusion in 8% (n=4), and hepatomegaly was incidentally detected in another 8% (n=4). A small proportion (2%, n=1) demonstrated peripancreatic fluid collection on ultrasonography. Ultrasonographic findings of patients with AP are presented in Table 2.

**Table 2:** Ultrasonographic Findings in Patients With Acute Pancreatitis

Ultrasonographic Finding	Number of Patients	Percentage (%)
Bulky Pancreas	38	72%
Altered Echotexture	19	38%
Poor Acoustic Window	9	18%
Peripancreatic Inflammation	2	4%
Peripancreatic Fluid Collection	1	2%
Ascites	15	30%
Pleural Effusion	4	8%
Hepatomegaly	4	8%

CECT of the abdomen was performed in all 50 patients as part of the radiological evaluation of AP. The most frequent radiological finding was a bulky pancreas, observed in 94% (n=47) of cases, consistent with pancreatic inflammation and edema. Peripancreatic inflammation or fat stranding was identified in 86% (n=43) of patients, highlighting extensive local tissue involvement. Peripancreatic fluid collections were noted in 16% of patients, with 10% (n=5) having a single collection and 6% (n=3) demonstrating multiple collections. Pancreatic necrosis, an indicator of severe disease, was seen in 24% (n=12) of patients. Additionally, portal vein thrombosis was present in 4% (n=2), and enlarged lymph nodes, either peripancreatic or paracolic, were observed in 6% (n=3) (Table 3). 

**Table 3:** CECT Findings in Patients With Acute Pancreatitis CECT: contrast-enhanced computed tomography

CECT Finding	Number of Patients	Percentage (%)
Bulky Pancreas	47	94%
Peripancreatic Inflammation/Fat Stranding	43	86%
Single Peripancreatic Fluid Collection	5	10%
Multiple Peripancreatic Fluid Collections	3	6%
Pancreatic Necrosis	12	24%
Portal Vein Thrombosis	2	4%
Peripancreatic/Paracolic Lymphadenopathy	3	6%

BISAP scoring

The BISAP score was calculated for all patients at the time of admission using clinical and routine biochemical parameters. A BISAP score of ≥3, indicative of severe AP and increased risk of mortality, was observed in three patients (6%). The remaining 47 patients (94%) had a BISAP score <3, suggestive of mild disease. Among the three mortality cases in the study, one patient had a BISAP score ≥3, while the remaining two had a score <3. This finding underscores that while BISAP ≥3 is associated with severe disease and higher mortality risk, low scores do not completely rule out the possibility of adverse outcomes (Table 4).

**Table 4:** BISAP Score and Patient Outcomes BISAP: Bedside Index for Severity in Acute Pancreatitis

BISAP Score	Deaths (n)	Discharges (n)	Total (n)
<3	2	45	47
≥3	1	2	3
Total	3	47	50

CT severity index (CTSI)

The CTSI was calculated based on Balthazar’s grading system, and the extent of pancreatic necrosis observed on CECT was used to stratify disease severity among the study population. Among the 50 patients, 35 (70%) were classified as having mild acute pancreatitis (CTSI score 0-3), nine (18%) had moderate severity (score 4-6), and six patients (12%) were categorized as having severe acute pancreatitis (score 7-10). Of the three patients who died during hospitalization, two belonged to the severe CTSI group, and one to the moderate severity group. No deaths were reported among patients with mild CTSI scores, suggesting a strong correlation between higher CTSI and increased risk of mortality (Table 5). 

**Table 5:** CTSI Score and Patient Outcomes CTSI: CT severity index

CTSI Category	Deaths (n)	Discharges (n)	Total (n)
Mild (Score 0–3)	0	35	35
Moderate (Score 4–6)	1	8	9
Severe (Score 7–10)	2	4	6
Total	3	47	50

Complications, outcomes, and mortality analysis

Eighteen (36%) patients developed complications, most of which were identified through CECT. The most common complication was pancreatic necrosis, observed in 26% (n=13) of cases, followed by pseudocyst formation in 18% (n=9). Portal vein thrombosis was noted in 4% (n=2) of patients. Several systemic complications were also documented. AKI developed in one patient who subsequently required hemodialysis. Another patient with pre-existing chronic kidney disease (CKD) also underwent dialysis during the admission. ARDS was seen in one case, while emphysematous pancreatitis-characterized by gas formation within necrotic pancreatic tissue-was diagnosed via CT in another. One patient was newly diagnosed with diabetic ketoacidosis (DKA) during the course of illness. Additionally, gastrointestinal bleeding manifestations such as hematemesis and melena were noted in one patient each. Surgical intervention, specifically cholecystectomy, was advised in patients with gallstone-related or biliary sludge-associated pancreatitis. One patient underwent necrosectomy due to extensive pancreatic necrosis.

Out of the 50 patients enrolled in the study, 47 (94%) were successfully discharged, while three (6%) succumbed to the illness during the first week of hospitalization. The mean duration of hospital stay was 9.6 ± 4.6 days (range: 5-25 days). Notably, one of the deceased patients had the shortest duration of stay (two days), while the longest hospital stay (25 days) was recorded in a patient who underwent surgical necrosectomy for pancreatic necrosis. Laboratory and prognostic scoring values were reviewed for the three patients who died. All three had elevated serum amylase and lipase levels, exceeding the cohort mean values. Two of the deceased patients had severe CTSI scores (7-10), and one had a BISAP score ≥3, and one deceased patient with a normal BISAP score and CTSI had high triglycerides. These findings support the association between higher BISAP/CTSI scores and increased mortality risk (Table 6).

**Table 6:** Laboratory and Prognostic Scores in Deceased Patients BISAP: Bedside Index for Severity in Acute Pancreatitis; BUN: blood urea nitrogen; CTSI: CT severity index; RBS: random blood sugar; TLC: total leukocyte count;

Patient	S. Amylase, IU/L	S. Lipase, IU/L	RBS, mg/dL	BUN, mg/dL	S. Creatinine, mg/dL	T. Bilirubin, mg/dL	TLC, cells/cumm	Triglycerides, mg/dL	S. Calcium, mg/dL	BISAP	CTSI
P-1	1342	926	156	15.87	1.2	1.6	8700	196	8.5	2	7
P-2	1126	1532	111	17.73	2.1	2.1	11,200	145	7.8	3	7
P-3	682	786	130	10.27	0.9	0.9	7600	266	8.2	2	2
Mean	606	617	113	18.25	1.3	1.4	8471	140	8.7	–	–

Prognostic utility and statistical evaluation of BISAP and CTSI

A BISAP score ≥3 was significantly associated with increased mortality (p=0.03977), highlighting its value as an early predictor of death in AP. However, its association with complications was not statistically significant (p=0.2537), indicating limited utility in predicting the development of complications (Table 7).

**Table 7:** Association of BISAP Score With Mortality and Complications in Acute Pancreatitis Mortality comparison: chi-square test (χ²=4.23, p=0.03977) - *statistically significant at p<0.05. Complications comparison: chi-square test (χ²=1.30, p=0.2537) - not statistically significant BISAP: Bedside Index for Severity in Acute Pancreatitis

BISAP Score	Deaths (n)	Survivors (n)	Test statistic (χ²)	P-value	Complications Present (n)	No Complications (n)	Test statistic (χ²)	P-value
≥3	1	2	χ²=4.23	0.03977^*^	2	1	χ²=1.30	0.2537
<3	2	45			16	31		
Total	3	47			18	32		

The CTSI demonstrated strong predictive value for both mortality and complications in AP. Among patients with severe CTSI scores (≥7), 33.3% (2/6) died, compared to only 2.3% (1/44) among those with mild to moderate CTSI scores (0-6), a statistically significant difference (χ²=9.039, p=0.00265). Furthermore, all patients with severe CTSI developed complications (100%), whereas only 27.2% (12/44) of those with mild to moderate scores had complications, again showing a statistically significant association (Yates χ²=9.17, p=0.00246). These results confirm the efficacy of CTSI as a robust predictor of both poor outcomes and disease severity in AP (Table 8).

**Table 8:** Association of CTSI With Mortality and Complications in Acute Pancreatitis Mortality comparison: chi-square test (χ²=9.03, p=0.00265) - ^*^statistically significant at p<0.05. Complications comparison: Yates-corrected chi-square test (χ²=9.17, p=0.00246) - *statistically significant at p<0.05 CTSI: CT severity index

CTSI Category	Deaths, n (%)	Survivors, n (%)	Test Statistic (χ²)	P-value	Complications Present, n (%)	No Complications, n (%)	Test Statistic (χ²)	P-value
Severe (7–10)	2 (33.3%)	4 (66.7%)	χ²=9.03	0.00265^*^	6 (100%)	0 (0%)	χ²=9.17	0.00246^*^
Mild–Moderate (0–6)	1 (2.3%)	43 (97.7%)			12 (27.2%)	32 (72.8%)		
Total	3	47			18	32		

**Figure 1:**
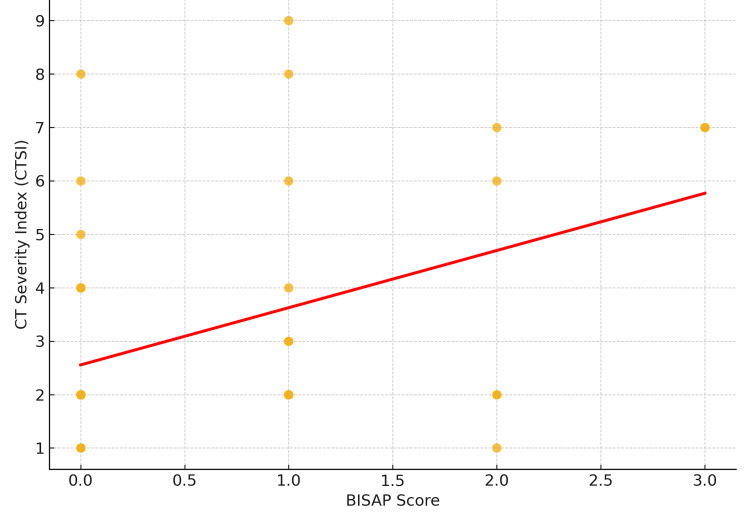
Correlation Between BISAP score and CTSI (Pearson r=0.45, p=0.019) BISAP: Bedside Index for Severity in Acute Pancreatitis; CTSI: CT severity index

When comparing BISAP and CTSI, a moderate positive correlation was observed (Pearson r=0.45, p=0.019), indicating that higher BISAP scores were associated with greater radiological severity as measured by CTSI. Of note, BISAP scoring offers practical advantages in settings where CT imaging is not immediately available, enabling early bedside risk stratification. However, CTSI remains superior in predicting the need for radiological or surgical interventions during the disease course (Figure 1). 

The ROC analysis demonstrated that the BISAP score had excellent predictive value for mortality in AP patients, with an area under the curve (AUC) of 0.93 (95% CI: 0.95-1.00). In contrast, the CTSI showed a moderate predictive value with an AUC of approximately 0.66 (95% CI: 0.56-0.98). BISAP, therefore, outperformed CTSI in mortality prediction in this group. In terms of diagnostic performance, both BISAP and CTSI demonstrated high specificity and negative predictive values (NPV), indicating that patients with low scores were unlikely to experience mortality. Notably, CTSI exhibited higher sensitivity (66.67% vs. 33.33%) and a superior negative likelihood ratio (0.35 vs. 0.6), suggesting that it is more effective in ruling out high-risk patients. In contrast, BISAP had slightly higher specificity (95.74%) and a strong positive likelihood ratio (8.3), reinforcing its value as a bedside tool for early identification of patients at elevated risk of death. However, the low sensitivity of BISAP indicates that a subset of patients with severe disease may be under-classified by relying solely on this score. These findings emphasize the complementary roles of both scoring systems: while BISAP is highly practical for early triage, CTSI remains critical for comprehensive assessment and guiding therapeutic decisions (Table 9).

**Table 9:** Predictive Performance of BISAP Score and CTSI for Mortality in Acute Pancreatitis BISAP: Bedside Index for Severity in Acute Pancreatitis; CTSI: CT severity index

Performance Metric	BISAP Score	CTSI
Sensitivity	33.33%	66.67%
Specificity	95.74%	91.48%
Positive Predictive Value (PPV)	33.33%	33.33%
Negative Predictive Value (NPV)	95.74%	97.72%
Positive Likelihood Ratio (PLR)	8.3	10.45
Negative Likelihood Ratio (NLR)	0.6	0.35

## Discussion

Rapid and accurate diagnosis, appropriate severity grading, and timely management remain central to improving outcomes in patients with AP. Identifying those at risk of severe disease through validated prognostic tools enables early intervention and intensive care when required. In the present study, we examined the clinical profile, laboratory and imaging findings, and outcomes of 50 patients with AP treated at a tertiary care center over two months. The mean age of our patients was 38 ± 11 years, with a predominance of cases (56%) in the 20-39 year age group, consistent with earlier Indian studies [14-16]. A striking male predominance was observed, with 90% of patients being male (M:F ratio of 9:1). This reflects the higher prevalence of alcohol consumption among men in rural India and is in line with prior Indian data [14-17].

Alcohol emerged as the most frequent etiological factor, implicated in 74% of cases, consistent with previous Indian studies where alcohol-associated AP remains predominant [14-17]. Gallstones were the second most common cause (6%), primarily among females, followed by idiopathic pancreatitis (10%) and drug-induced pancreatitis (4%). These findings reaffirm alcohol and gallstones as the leading causes of AP, though proportions vary globally. Prolonged alcohol consumption, as established in the literature, continues to contribute significantly to disease burden and recurrence in this population [14-17]. The clinical presentation in our study population mirrored classical descriptions of AP. Abdominal pain was the most universal symptom (98%), accompanied by nausea (68%) and vomiting (62%).

Systemic manifestations, including fever (10%) and jaundice (12%), reflected either systemic inflammatory response syndrome (SIRS) or underlying hepatobiliary disease. Breathlessness (12%) and decreased urine output (8%) were observed in a subset of patients and heralded the onset of serious complications such as ARDS and AKI, necessitating vigilant monitoring and supportive care. Ultrasound served as a useful initial imaging modality but demonstrated limitations in detecting subtle or deep pancreatic changes. CECT provided superior anatomical detail and was critical for assessing complications and guiding management decisions. Our findings align with existing evidence that CECT remains the gold standard for evaluating structural pancreatic injury [6,7]. The emerging role of diffusion-weighted MRI, with its non-ionizing profile and ability to detect early parenchymal changes, holds promise as an adjunct or alternative modality, particularly in patients with renal impairment [6,7].

Recurrent acute pancreatitis (RAP) was documented in 20% of patients, consistent with the recurrence rates reported in prior studies [14-17]. Lack of alcohol abstinence and inadequate surgical management of gallstone-related pancreatitis were key contributors to RAP. Recurrence was more common among alcohol-associated AP cases, echoing existing literature on the high recurrence risk linked to continued alcohol use [18]. Comorbidities such as diabetes mellitus (6%), hypertension (14%), and CKD (4%) were frequently encountered and can potentially impact disease severity and complicate management. The application of BISAP scoring and CTSI provided valuable prognostic insights; 6% of patients had BISAP scores ≥3, correlating with greater risk of mortality and complications. Similarly, 12% of patients had severe CTSI scores (7-10), among whom mortality was concentrated.

Our findings emphasize the reliability of combining clinical and radiological scoring for early risk stratification, supporting the utility of BISAP and CTSI in routine practice [19,20]. Overall mortality in our study was 6%, with alcohol-associated severe AP accounting for the majority of deaths. Pancreatic necrosis was observed in 26% of patients and was strongly associated with worse outcomes. Complications such as ARDS, AKI, and portal vein thrombosis were documented, particularly among patients with high CTSI scores and necrotizing pancreatitis. The high burden of complications among alcohol-related AP cases further highlights the need for aggressive supportive management and long-term preventive strategies. Additionally, in patients with alcohol-related AP, the presence of alcohol withdrawal can further increase clinical risk, with reported mortality rates ranging from 1% to 4% [16,17,21].

This study has several limitations. It was conducted at a single tertiary care teaching hospital and involved a relatively small sample size of 50 patients, which may limit the generalizability of the findings. The short follow-up duration restricted long-term outcome assessment. BISAP scoring was applied at admission, and dynamic changes during hospitalization were not captured. Radiological interpretation of CTSI may have observer variability. Additionally, laboratory and imaging facilities may not be uniformly available in rural or primary healthcare settings, limiting external applicability. Despite these limitations, the study provides valuable insights into the prognostic utility of BISAP and CTSI scores in AP within a resource-constrained clinical environment.

## Conclusions

This study provides valuable insights into the etiopathogenesis, clinical profile, and outcomes of AP in a tertiary care setting. Alcohol was identified as the predominant etiological factor, contributing to both disease severity and complications. The BISAP score emerged as an effective, practical tool for early mortality risk stratification, while CTSI offered superior accuracy in predicting complications and guiding interventional management. A moderate positive correlation between BISAP and CTSI underscores their complementary utility. The combined application of these scoring systems can enhance clinical decision-making, especially in settings with limited resources, ultimately contributing to improved patient outcomes in AP.
